# An integrated genomic and metabolomic framework for cell wall biology in rice

**DOI:** 10.1186/1471-2164-15-596

**Published:** 2014-07-15

**Authors:** Kai Guo, Weihua Zou, Yongqing Feng, Mingliang Zhang, Jing Zhang, Fen Tu, Guosheng Xie, Lingqiang Wang, Yangting Wang, Sebastian Klie, Staffan Persson, Liangcai Peng

**Affiliations:** National Key Laboratory of Crop Genetic Improvement, Huazhong Agricultural University, Wuhan, Hubei 430070 P. R. China; Biomass and Bioenergy Research Centre, Huazhong Agricultural University, Wuhan, Hubei 430070 P. R. China; College of Plant Science and Technology, Huazhong Agricultural University, Wuhan, Hubei 430070 P. R. China; College of Life Science and Technology, Huazhong Agricultural University, Wuhan, Hubei 430070 P. R. China; Max-Planck-Institute for Molecular Plant Physiology, Am Mühlenberg 1, 14476 Potsdam, Germany; School of Botany, University of Melbourne, Melbourne, VIC 3010 Australia

**Keywords:** Rice, Cell wall, Co-expression network, Metabolomics

## Abstract

**Background:**

Plant cell walls are complex structures that full-fill many diverse functions during plant growth and development. It is therefore not surprising that thousands of gene products are involved in cell wall synthesis and maintenance. However, functional association for the majority of these gene products remains obscure. One useful approach to infer biological associations is via transcriptional coordination, or co-expression of genes. This approach has proved useful for several biological processes. Nevertheless, combining co-expression with other large-scale measurements may improve the biological inferences.

**Results:**

In this study, we used a combined approach of co-expression and cell wall metabolomics to obtain new insight into cell wall synthesis in rice. We initially created a weighted gene co-expression network from publicly available datasets, and then established a comprehensive cell wall dataset by determining cell wall compositions from 29 tissues that almost cover the whole life cycle of rice. We subsequently combined the datasets through the conversion of co-expressed gene modules into eigen-vectors, representing expression profiles for the genes in the modules, and performed comparative analyses against the cell wall contents. Here, we made three major discoveries. First, we confirmed our approach by finding primary and secondary wall cellulose biosynthesis modules, respectively. Second, we found co-expressed modules that strongly correlated with re-organization of the secondary cell walls and with modifications and degradation of hemicellulosic structures. Third, we inferred that at least one module is likely to play a regulatory role in the production of G-rich lignification.

**Conclusions:**

Here, we integrated transcriptomic associations and cell wall metabolism and found that certain co-expressed gene modules are positively correlated with distinct cell wall characteristics. We propose that combining multiple data-types, such as coordinated transcription and cell wall analyses, may be a useful approach to glean new insight into biological processes. The combination of multiple datasets, as illustrated here, can further improve the functional inferences that typically are generated via a single type of datasets. In addition, our data extend the typical co-expression approach to allow deeper insight into cell wall biology in rice.

**Electronic supplementary material:**

The online version of this article (doi:10.1186/1471-2164-15-596) contains supplementary material, which is available to authorized users.

## Background

Gene co-expression analyses can reveal functional relationships between gene products. These types of relationships are typically explored using some type of similarity measure, e.g. Pearson’s correlation coefficient (PCC), to quantify the association between two genes in the genome. The pairwise relationships can be represented as a network structure, in which edges (co-expression relationships) connect nodes (genes) that generally include the majority of genes in a given organism’s genome [1]. Based on these associations, it is possible to predict functional gene clusters, or groups of genes, that participate in common biological pathways [2, 3]. Moreover, this approach may also be used to find the conserved orthologous gene clusters across several species [4, 5], with the implication that the clusters are involved in similar biological processes.

Many co-expression networks have been constructed in plants, such as Arabidopsis [1, 3, 6–11], barley [12], rice [13, 14], poplar [15], tobacco [16], and maize [17]. Several of these efforts have been implemented as web-based tools, e.g. the Arabidopsis Co-expression Toolkit (ACT) [18], ATTED-II [19], AtCOECis [20], RiceArrayNet (PlantArraynet) [14], Co-expressed biological processes (CoP) database [15], The Gene Co-expression Network Browser [13], and two AraNets [1, 9], and PlaNet [21].

While the co-expression-based approaches have proved successful for several biological processes, far from all cellular aspects can rely on this type of metrics. Instead, integrative approaches are increasingly applied to extend knowledge gleaned from one type of dataset. These studies are typically relying on functional and structural genomics data, such as high-throughput microarray assays, next-generation sequencing, and metabolomic and proteomic technologies [22].

Plant cell walls are mainly composed of cellulose, non-cellulosic polysaccharides (hemicelluloses and pectin) and lignin, and represent the most abundant renewable biomass on earth [23]. The primary and secondary cell walls are typically distinct structures in plant growth and development [23], where the primary wall is a flexible matrix that allows directed cell growth and the secondary wall is a robust structure surrounding cells that need extra support for their functions. In general, cellulose makes up almost 25-30% of dry matter in grasses [24] and 40-45% in woody plants [25]. Hemicelluloses are polysaccharides that contain xyloglucans, xylans, mannans and glucomannans, and β-(1 → 3,1 → 4)-glucans, whereas pectins are diversified compounds that mainly are present in primary walls [26]. Lastly, the polyphenolic molecule lignin is an amorphous polymer of phenylpropane units with three monomers: *p*-hydroxyphenyl (H), guaiacyl (G), and syringyl (S) [27, 28], laid down during secondary wall formation.

More than one thousand gene products have been proposed to be dedicated to plant cell wall biogenesis and modification [29–31]. During the past years, characterization of plant cell wall mutants has revealed dozens of genes involved in cell wall synthesis and modification [28, 32–41]. However, the functions of the majority of cell wall related genes remain obscure, in particular in plant species other than the main model organism Arabidopsis.

Rice (*Oryza sativa*) is one of the most important food crops worldwide and serves as a model plant for cereal genomic research [42, 43]. Genomic resources in rice have developed rapidly in recent years, in part due to the high quality genome sequence. Genome-wide expression data of 39 tissue/organs covering the life cycle of rice has been obtained and deposited in the public database [44]. To date, most insights into cell wall related properties of rice have been obtained through the characterization of mechanical strength change in stems in forward genetic screens [45, 46]. These studies have revealed some mechanistic aspects of cell wall biosynthesis and identified the affected genes in rice [47]. However, genetic screens are usually quite involved, and a more rapid way of inferring relationships between gene products and cell wall characteristics would certainly be advantageous. In this study, we concentrated on establishing a platform that combines transcriptomic associations with cell wall characteristics to infer processes that are connected to cell wall biosynthesis and re-modeling in rice.

## Results and discussion

### Data integration and network construction

To construct the rice gene co-expression network, microarray datasets from 39 tissues, covering almost the whole life cycle of rice, were initially collected from CREP (Collections of Rice Expression Profiling, http://crep.ncpgr.cn). After data quality control and removal of non-specific binding probes, a total of 38,868 probe sets were used for mapping to the TIGR database using the Nipponbare genome sequence as reference. This resulted in 31,574 probe sets each having at least six perfect match probes mapped as unique genes, and thus termed by the corresponding genes. As expression for any given gene is measured by multiple probes (Additional file 1), we summarized the information using R function *collapseRows* [48, 49]. The resulting expression matrix contained 33,204 genes or probe sets. To be able to statistically compare the expression matrix to the cell wall data, we decided to construct a weighted correlation network [50] based on the 33,204 probes for the 29 tissues that we also used for cell wall analyses. Here, the weights of edges in the corresponding co-expression network correspond to the degree of similarity of the expression profiles of two adjacent nodes/genes.

Subsequently, a clustering approach of the weighted correlation network was undertaken, which resulted in 56 groups of highly co-expressed genes, also referred to as gene modules (Additional file 2). Hence, modules were defined as groups of genes which exhibit a high intra-module topological overlap [51]. The modules were denoted by numbers from zero to 55 and prefixed with “ME” referring to “module eigengene” [50]. Obviously, the numbers of genes (probe sets) per module differed, and more than half of the modules contained less than 500 genes (probe sets) (Additional file 3A). To explore the co-expression relationships between modules, a module’s representative expression pattern was summarized using the first principal component of all the module’s gene members. Further, all module eigengenes were clustered by using complete linkage method (Additional file 3B), which characterizes the similarity structure between the modules.

### Biological relevance and connectivity scores of network modules

To assess the functional relevance of the gene modules, and to make sure that the co-expressed modules reflect biologically relevant information, we next examined whether certain ontology terms were over-represented in the modules. Gene ontology (GO) enrichment analysis was therefore performed using a weighted method and Fisher’s exact test [52] (Additional file 4). The analysis showed that a total of 4,014 enriched terms and 1,175 unique terms were identified among the modules at *p* < 0.05. Notably, a significant over-representation of the terms cellulose and non-cellulosic polysaccharide biosynthesis was observed for Module 24 (with 406 genes or probe sets) and Module 44 (with 136 genes or probe sets) (Additional file 4). Based on the representation of KEGG reference pathway maps and BRITE functional hierarchies [53], we furthermore performed a functional enrichment analysis of KEGG Orthology for each module using hypergeometric tests. Module 24 and Module 44 were enriched in glycan biosynthesis and metabolism, consistent with the findings that genes in Module 24 and Module 44 may participate in cellulose and non-cellulosic biosynthesis as observed in the GO enrichment analysis. Detailed significant associations for each module are supplied in Additional file 5.

Highly connected genes, or hubs, are thought to play a central role in biological networks. Connectivity has been found as an important complementary gene screening variable for finding biologically significant genes in particular biological processes [54]. Intramodular connectivity (kWithin) is defined as the gene connectivity inside a given module. In weighted networks, intramodular connectivity equals the sum of connection weights of a node with all other nodes inside module. In this study, we defined whole network connectivity kTotal, and external module connectivity (kOut = kTotal-kWithin) for any given node. To find genes of high connectivity (i.e. 'hubs’) in consensus modules, we evaluated the module eigengene-based connectivity (kME) as the correlation between the gene expression and the module eigengene [55]. We also calculated all connectivity types in all models, and the genes sorted out by their kME were listed in Additional file 6.

### Cell wall composition analysis

**Figure 1:**
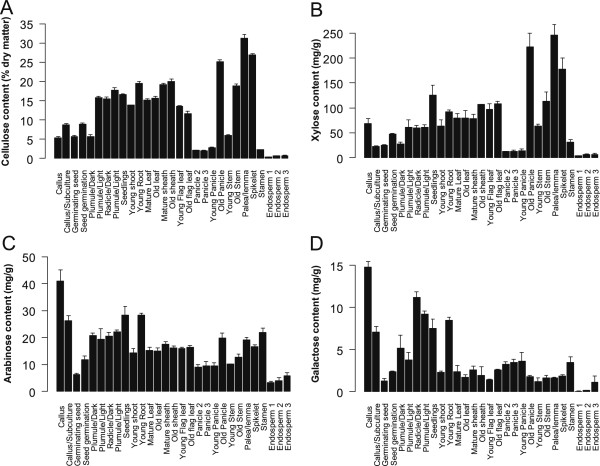
**Cell wall content varies across rice tissues. A**. to **D**. Cellulose and three monosaccharides (xylose, arabinose and galactose) of hemicelluloses were estimated in 29 rice tissues.

**Figure 2:**
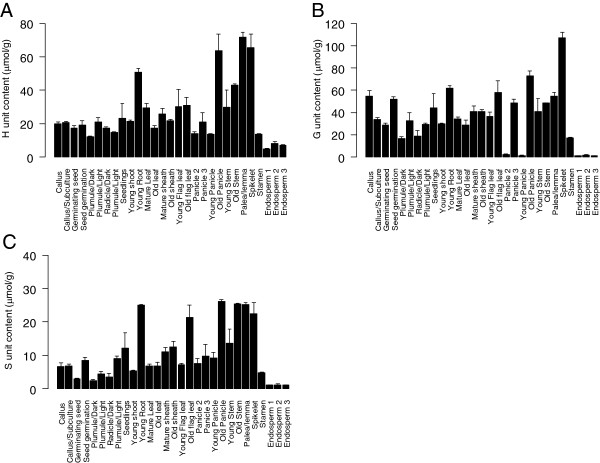
**Monolignol content across rice tissues.** Three monolignols of 29 tissues in rice were measured. These were *p*-hydroxybenzaldehyde (H), vanillin (G) and syringaldehyde (S).

In an attempt to assign certain cell wall related functions to the modules, we harvested material from the 29 tissues that corresponded to the microarray data sets above. We sequentially extracted wall polysaccharides including pectin with ammonium oxalate, hemicelluloses with KOH, and cellulose in the remaining pellet [56, 57]. The pectin was present at very low levels, or absent, in most rice tissues, and we therefore did not use the pectin data for any further investigation in this work. In summary, the cell wall composition varied greatly across the 29 tissues (Figure 1; Additional file 7). Cellulose content ranged from 0.29% (endosperm1) to 31.33% of dry matter (palea/lemma) (Figure 1A). Three major monosaccharides of hemicelluloses also varied significantly [58], with xylose (Xyl) levels ranging from 3.49 (endosperm1) to 245.82 mg/g (palea/lemma), arabinose (Ara) levels ranging from 3.26 (endosperm1) to 41.07 mg/g (callus), and galactose (Gal) levels ranging from 0.03 (endosperm1) to 14.81 mg/g (callus) (Figure 1B,C,D; Additional file 7). The main constituents of lignin, i.e. the H, G and S monolignols, also showed major changes among tissues; *p*-hydroxyphenyl (H) levels varied from 4.93 (endosperm1) to 71.72 μmol/g (palea/lemma), guaiacyl (G) ranged from 1.18 (endosperm1) to 107.19 μmol/g (spikelet) and syringyl (S) ranged from 1.06 (endosperm2) to 25.42 μmol/g (old stem) (Figure 2; Additional file 7). Hence, the plant cell wall composition displayed major differences across the different rice tissues.

To assess the degree of correlation of the cell wall components across the tissues, we performed a correlation analysis of the glucose of cellulose, monosaccharides of hemicelluloses and monomers of lignin (Additional file 8). Interestingly, cellulose was significantly positively correlated with Xyl (0.89) and the three monolignols (H: 0.81, S: 0.70 and G: 0.71) with student asymptotic *p*-value at *p* < 0.01, whereas Ara and Gal exhibited a significant positive correlation (0.81) at *p* < 0.01. Notably, the three monolignols were also positively correlated to each other (0.81, 0.89 and 0.76) at *p* < 0.01. These data suggest that certain cell wall components are produced in a coordinated fashion in rice.

### Connecting module eigenvectors and cell wall datasets

**Figure 3:**
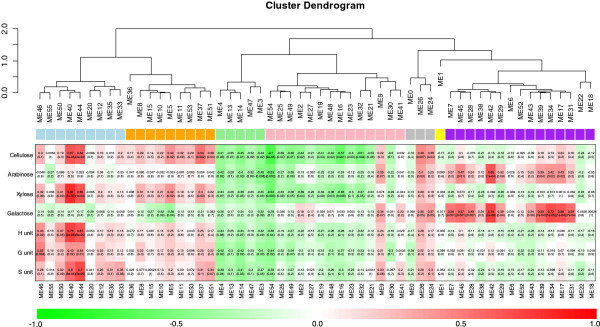
**Certain modules correlate with specific cell wall content in rice.** Correlative analysis between cell wall composition and module eigenvectors. Boxes contain Pearson correlation coefficients and their associated P values. Color scales indicated the correlation coefficient levels: red and green for high and low correlation level, respectively. The hierarchical clusters of Module eigenvector were obtained using the agglomeration method of 'complete linkage’ based on the Euclidean distances of all Module eigenvector similarities in cell wall characteristics. The clades are, furthermore, color coded (colored lines above the clades). These colors are used to denote cell wall characteristics in the module based co-expression network depicted in Figure 4.

To investigate the associations between the co-expressed gene modules and the cell wall composition we conducted correlation analyses between the previously derived module representative eigengenes and the cell wall composition data using PCC (Figure 3).

**Figure 4:**
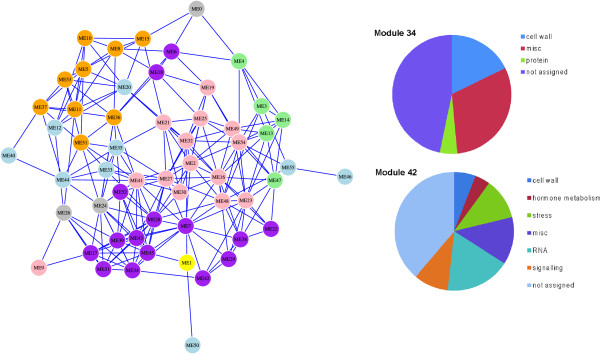
**Rice module-related co-expression network, and MapMan ontology term enrichment for Modules 34 and 42.** (Left panel) Module based gene co-expression network in rice. Different colors of the nodes (modules) indicate different cell wall composition associated with the module eigenvectors. Colors as indicated in Figure 3 (clade color code). (Right panel) Pie charts depicting ontology term enrichment for genes in Modules 34 (upper pie) and 42 (lower pie). Only major Bin terms have been used for pie construction. For complete set, see Additional file 9A and B. Only ontology terms with a significance score above 0.001 are shown.

A cell wall related process that has extensively been used for co-expression analyses is secondary wall synthesis [59]. As this process also should be associated with distinct cell wall characteristics, i.e. cellulose, xylan and lignin, we first investigated whether such patterns were evident from our cell wall analyses, and secondly, if these characteristics correlated positively with any modules. Notably, Module 44 displayed a significantly positive correlation with cellulose, xylose, and H and S monolignols (*p* < 0.001), suggesting that the module plays a major role in the synthesis of these components. Strikingly, the genes contained in Module 44 included cellulose synthases (*CesA*s), *OsCOBRA*, chitinase-like (*CTL*) and other genes that have been assigned to cellulose biosynthesis (Additional file 6A) [42]. Furthermore, several of the genes included in this module have been obtained via forward genetic screens for brittle culms, such as *OsBC1, OsBC7, OsBC11* [45, 47, 60, 61]. Based on these observations, and the fact that the closest homologs for many of the genes in this module are involved in secondary wall formation in Arabidopsis, we assumed that Module 44 is associated with secondary wall synthesis. These data were supported by the fact that the rice orthologs of the NAC transcription factor (TF) *SND2* (*LOC_Os05g48850*) and the MYB TFs, *AtMYB42* (*LOC_Os09g36250*), *AtMYB52/54* (*LOC_Os03g51110*), *AtMYB58/63* (*LOC_Os04g50770, LOC_Os02g46780*) and *AtMYB103* (*LOC_Os08g05520*) were included in this module. These TFs have been shown to regulate secondary wall deposition in Arabidopsis [62–64]. Surprisingly, the two well-known secondary wall TFs (*OsSWN2/LOC_Os08g02300*, *OsSWN1/LOC_Os06g04090*) were found in modules 21 and 51. Although these modules did not show any significant correlation with cell wall polymers in this study, both modules are in close vicinity of modules 24 and 44 (Figure 4; i.e. somewhat co-expressed with these modules) indicative of their role in cell wall formation [65, 66]. Several genes were identified for glucuronoxylan biosynthesis, such as the rice homologs for *AtIRX14L* (*LOC_Os06g47340*, GT43 family, kME 0.776) [67], *AtIRX15L* (*LOC_Os04g55640*, DUF 579, kME 0.717) [68], and the rice xylosyltransferase *OsGT61-1* (*LOC_Os02g22380*, kME 0.714) [69] in module 44. The strong positive correlation between the module eigengene and the cell wall content, which typified secondary wall content, supports that our combined large-scale dataset strategy is useful in obtaining meaningful biological information.In an attempt to assess how the cell wall characteristics were globally distributed over the co-expressed modules, we color-coded the cell wall-related clades obtained in the dendrogram in Figure 3 (see clade coloration in this figure). Figure 4 shows that the different cell wall characteristics nicely groups together in a module meta-network, i.e. a network that displays the co-expression relationships between the modules. These data corroborate that certain groups of co-expressed modules may be linked to distinct cell wall features. For example, the purple well-grouped modules typically correlate with high levels of galactose and arabinose (compare Figures 3 and 4).

### Module 24 genes participate in primary cell wall cellulose formation

Based on the GO enrichment analysis, Module 24 was also identified as likely to be associated with cellulose biosynthesis (Additional file 4; Figures 3 and 4). Notably, many genes for primary wall cellulose biosynthesis were included in this module (Additional file 6B), such as *OsCESA1, 3, 5, 8, OsCSLF6* and *OsCOBRA* [42]. Interestingly, orthologous genes to the once associated with xylan backbone elongation in Arabidopsis [70, 71] were also identified in Module 24, including *AtIRX9* (*LOC_Os05g03174*, GT43 family), *AtIRX9L* (*LOC_Os01g48440*, GT43 family), *AtIRX10* (*LOC_Os01g70190/LOC_Os01g70200/LOC_Os10g10080*, GT47 family) and *AtIRX14* (*LOC_Os04g55670*, GT43 family). Here, it is important to bear in mind that the rice primary walls, in contrast to Arabidopsis, contain large amounts of glucuronoarabinoxylan. Curiously, four lignin biosynthesis related genes were also found in this module, i.e. *OsCCR1/IRX4* (*LOC_Os08g34280*) [72], *OsCCOMT* (*LOC_Os08g38900*), *Os4CL1* (*LOC_Os06g44620*), and *OsCOMT* (*LOC_Os08g06100*). One possible explanation for this is that the co-expressed gene vicinities for primary and secondary wall synthesis are more closely connected in monocots than in dicots (observations made from PlaNet) [21]. Hence, it is plausible that the lignin related genes in module 24, which represent rather early steps in the lignin biosynthesis, represent connecting elements between the primary and secondary wall production. Importantly, Arabidopsis homologs for several TFs contained in Module 24 have been reported to regulate plant cell wall formation in *Arabidopsis* [73–76], including *AtMYB86* (*LOC_Os05g46610*), *AtMYB61* (*LOC_Os07g44090*), *ANAC073/SND2* (*LOC_Os01g48130*) and *AtVND4* (*LOC_Os02g42970*). In addition, *LOC_Os03g42630* and *LOC_Os04g43560* are also present in this module and correspond most closely with *ANAC058* and *ANAC074* in Arabidopsis. Although, these TFs have not been associated with cell wall formation previously they may represent interesting candidates for such functions. Intriguingly, several of the TFs in this module typically regulate features associated with secondary wall synthesis, corroborating a close connection between primary and secondary wall synthesis in grasses. As Module 24 is significantly correlated with cellulose and other non-cellulosic cell wall monomers, and given that several orthologs correlate with primary wall cellulose synthesis in *Arabidopsis*, we conclude that Module 24 is enriched for primary wall cellulose related functions.

### Module association with other cell wall characteristics

The cell wall content and gene co-expression supported a role for the genes in Modules 24 and 44 in cellulose synthesis during primary and secondary wall synthesis, respectively. These findings are consistent with previous findings [5]. This also suggests that the cell wall characteristics for a module may be complementary to the co-expression approach to assign function(s) for the module. We therefore argued that other modules that have tight positive correlation with certain cell wall characteristics (Figure 3) can be assigned to such functions with the additional aid of co-expression. Several modules are strongly associated with specific cell wall characteristics, including Modules 7, 17, 34, 37, 39, 40, and 42. Module 34 has a strong positive correlation with Ara and Gal cell wall content (Figure 3), but little is known about the function of the genes associated with this Module. To first investigate whether the genes included in the Module are associated with cell wall synthesis or modifications, we analyzed the genes for MapMan ontology term associations [77]. Figure 4 shows a pie chart of significant MapMan terms associated with Module 34. Interestingly, the term cell wall was among the most highly enriched terms for the genes included in the Module. More specifically, the term hemicellulose synthesis was highly significantly enriched (*p*-value < 3.6e-17) as was the term cell wall modification (*p*-value < 6.4e-12; Additional file 9A). These terms corresponds very well with the positive correlation of the Module with Gal and Ara content, which are typically found associated with hemicelluloses in grass species. Moreover, when we investigated the annotation of the genes included in Module 34, we discovered that many of the genes were associated with glycosyltransferase and other cell wall annotation, including expansin (*LOC_Os10g30340, LOC_Os01g14650, LOC_Os02g16800, LOC_Os02g16780, LOC_Os02g16730, LOC_Os03g06010*), endoglucanse (*LOC_Os08g12800, LOC_Os11g14410, LOC_Os04g36610*) and peroxidase (*LOC_Os07g44550, LOC_Os08g42030, LOC_Os05g04410, LOC_Os03g02939, LOC_Os01g16450*) activities. More specifically, we found that many xyloglucan associated functions, including xyloglucan galactosyltransferase (*LOC_Os10g32170, LOC_Os07g09670*), fucosyltransferase (*LOC_Os09g28460, LOC_Os02g52630, LOC_Os08g24750, LOC_Os06g10910*), and CSLC3-cellulose synthase-like (*LOC_Os08g15420*) families, where present in the Module. While xyloglucan is not a prominent wall component in grasses, it is likely that these genes play important functions in hemicellulose synthesis in these species. Also, as Ara and Gal are two major monosaccharides branched in xylans in grasses [26, 57, 58], the Ara and Gal substitution degrees could be determinants in hemicellulosic structures. In addition, expansins are typically associated with modifying interactions between hemicelluloses and cellulose, and therefore induce extensibility in the wall matrix. Hence, it appears likely that many of the components included in Module 34 would have a direct influence on the formation and modification of hemicellulose polymers and interactions.

Module 42 was, similar to Module 34, also significantly positively correlated with Ara and Gal content (Figure 3). Similar to analyses done for Module 34, we investigated the genes in Module 42 for significant MapMan ontology enrichments (Figure 4; Additional file 9B). From this analysis it became evident that terms like cell wall degradation was prominent (*p*-value < 1.4e-05). In particular, many glycosyl hydrolases, such as *OsGH3* (*LOC_Os02g51620*), *OsGH16* (*LOC_Os10g39840*), *OsGH9* (*LOC_Os01g12070/LOC_Os02g03120*), *OsGH17* (*LOC_Os03g62860*) and *OsGH31* (*LOC_Os01g03950*) are part of this Module. Similar to Module 34, we also found several expansin genes, including *LOC_Os05g15690*, *LOC_Os07g29290*, and *LOC_Os10g40720*. These data support a role in cell wall modification and degradation for Module 42. To analyze the associations between the Module components and the cell wall content a bit more in detail, we calculated PCC between the genes, or probes, with the cell wall components and student asymptotic *p*-values. The more likely candidate genes were sorted out based on the correlative coefficient *p* values relatively lower than 75% of all student asymptotic *p*-values according to cell wall component. As a result, a subgroup of 13 genes from Module 42 showed a high correlation with Ara level and another subgroup of 13 genes correlated significantly with Gal content (Additional file 6D). Notably, another 32 genes in Module 42 that were not part of the two previous subgroups displayed a correlation with both Ara and Gal (Ara + Gal) levels. Notably, several OsGH9 family members have recently been identified with a role in cellulose crystallinity modification [43], and the Ara substitution degree of xylans in *Miscanthus* displays a significantly negative correlation with the cellulose crystalline index [58]. Perhaps these relationships are also reflected in the Module 42 correlation with Ara. Taken together, it is plausible that the genes in Module 42 could be associated with cell wall modification and degradation of cell wall polymers, particularly concerning hemicelluloses. While the Module included several cell wall related genes, most notably glucanses, most of the other genes have not previously been associated with cell wall related functions. We find it likely that these genes might reflect underlying growth programs that need to be coordinated with cell wall modifications. These relationships will certainly be interesting to explore in more detail in the future.

Module 40 displayed a significantly positive correlation with cellulose, Xyl, and H or S, which is similar to Module 44 (Figure 3). However, the majority of genes contained in this module have not been associated with cell wall synthesis or modification yet. To investigate the potential function in cell wall formation process of genes or probes included in this Module, we also performed a correlation analysis between the genes or probes with the cell wall components (cellulose, Xyl, H, S) and calculated the student asymptotic *p*-values. We then sorted the *p*-values calculated between genes or probes expression values with the cell wall components, individually. Genes or probes got lower *p*-values than 75% of all student asymptotic *p*-values calculated were screened out as candidate genes according to the cell wall components. The selected genes were then classified into ten groups. A subgroup of six genes exhibited a high correlation with cellulose, while another subgroup that held seven genes correlated significantly with xylose (Additional file 6C). Another four genes that were not found in the previous subgroups exhibited a correlation with both cellulose and Xyl levels (Cel + Xyl), suggesting a potential role for the corresponding gene products in wall synthesis or modification. In addition, a NAC TF (*LOC_Os03g01870*) was associated with subgroup Multi which may play multiple roles in cell wall synthesis or modification. As NAC TFs have been reported as key regulators of secondary cell wall synthesis in *Arabidopsis* [64], we propose that the *OsNAC* gene may similarly play a regulatory role in the cell wall networks associated with Module 40. Hence, our data suggested that Module 40 may participate in re-organization of the cell wall.

Furthermore, 32 genes in module 46 were highly correlated with G-monolignols based on the student asymptotic *p*-values calculated between the G content with the expression values of genes, or probes (Figure 3; Additional file 6E). However, none of these genes encode the enzymes involved in the lignin biosynthesis pathways [27]. Notably, the *OsMYB26* TF (*LOC_Os01g51260*) of this module corresponds to the *Arabidopsis* MYB TF *AT3G13890* (also known as *AtMYB26*) (Additional file 6E). This TF has been identified as an activator of secondary wall thickening [78, 79]. Hence, we hypothesize that Module 46 may be involved in the regulation of secondary cell walls, in particular for the production of G-rich lignification.

To further find more complex associations between gene modules represented by their *eigengenes* and cell wall properties, we used canonical correlation analysis (CCA). CCA is a multivariate statistical technique employed for studying associations between two sets of variables [80], which are represented as two matrices X and Y. Instead of analyzing pair-wise similarities of *individual variables* as it is the case for Pearson’s correlation coefficient, CCA finds two linear combinations for each of the *two matrices* X and Y, which are maximally correlated and was used here to find association between cell wall related measurements and modules of transcripts represented by eigengenes.

**Figure 5:**
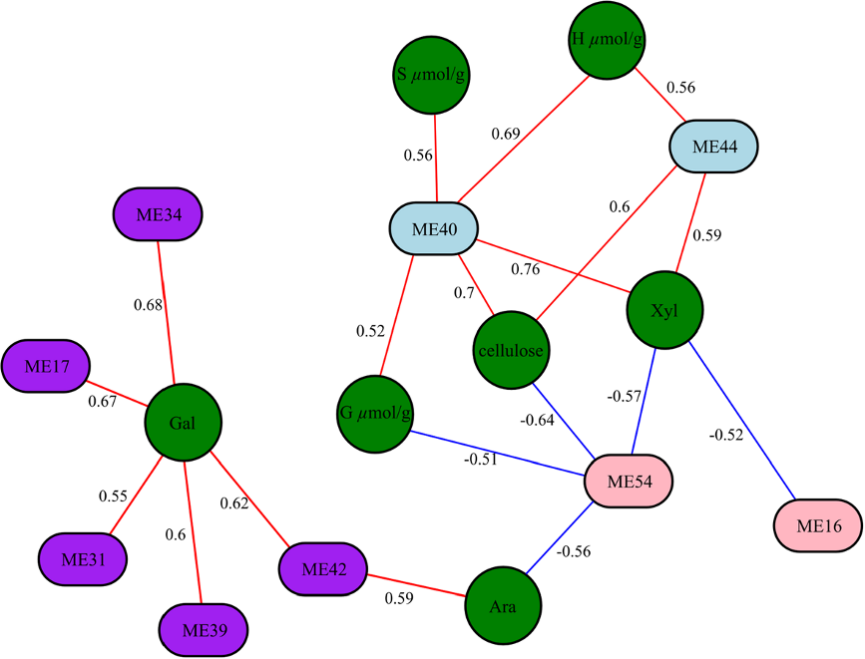
**A model on the module network for cell wall metabolomics.** Central modules 44 exhibited a dominant function on cellulose biosynthesis from primary to secondary cell wall formation, followed with the module 40 involved in re-organization of cell wall; Modules 34 and 42 displayed a distinct modification in hemicellulosic formation and degradation; Cell wall related features are represented as circles, while eigengenes are depicted by (rounded) rectangles. An edge between a circle and a hexagon illustrates an association found by CCA (blue colored edges show negative, and red colored edges, positive association, respectively).

Clear similarities resulting from the CCA between eigengenes and cell wall properties are visualized in a relevance network (Figure 5; *cf.* Methods). Here, cell wall related features are represented as circles, while eigengenes are depicted by (rounded) rectangles. An edge between a circle and a hexagon illustrates an association found by CCA (blue colored edges show negative, and red colored edges, positive association, respectively).Again, Modules 40 and 44 are clearly related to each other (compare cell wall characteristics and gene module co-expression network; Figures 3 and 4) and are associated with typical secondary wall traits, such as lignin monomers, cellulose and xylose (Figure 5). Several modules are also positively associated with galactose, including the modules 17, 31, 34, 39, and 42, perhaps indicative of some primary wall hemicellulose synthesis and plant growth as indicated above. Interestingly, module 54 showed a negative association with several cell wall traits, including xylose, arabinose, cellulose and G lignin monomers (Figures 3 and 5). This could perhaps indicate that the genes associated with this module negatively regulate the deposition of cell walls in general.

## Conclusions

We integrated transcriptomic associations and cell wall metabolism for 29 rice tissues and found that certain co-expressed gene modules are positively correlated with distinct cell wall characteristics. In addition to confirmatory relationships, i.e. that primary and secondary wall gene modules were correlated with cellulose synthesis, cell wall related characteristics for several other modules could be inferred. Based on these relationships we propose a draft network for cell wall metabolomics (Figure 5). This framework may lie as a foundation for cell wall transcriptional regulatory and biosynthesis network in monocots and beyond. It is important to note that these inferences would have been difficult to achieve by simply looking at co-expression alone. We therefore propose that combining multiple data-types, such as coordinated transcription and cell wall analyses, may be a useful approach to glean new insight into biological processes.

## Methods

### Data integration and network construction

A total of 98 Affymetrix Rice GeneChip Genome Array microarray datasets were obtained from CREP (Collections of Rice Expression Profiling, http://crep.ncpgr.cn) from an *indica* variety (Zhenshan 97). This dataset comprises transcriptomics profiling of 39 different tissues (organs) covering the whole life cycle of rice. Note, that those samples which involve hormone treatments (NAA, GA3 and KA) and additional seven samples (Additional file 7) were discarded to match the tissues from which cell walls were measured. Before mapping all probe sets to the annotated rice gene models, those probe sets that exhibited expression values lower than 80% of the expression values for the negative control probe sets on the microarray in any of 39 tissues were removed and were assumed as background intensities (noise). This filtering step resulted in a total number of 38,868 probe sets for further analysis. Successively, the BLAST-like alignment tool (BLAT) developed by Kent [81] was used to align the nucleotide sequences of the remaining probe sets to the Michigan State University (MSU) Rice Genome Annotation version 6.0 [82] which currently contains 56,797 protein-coding gene models (BLAT parameters used: *minIdentity* = 100, *minMatch* = 1, *stepSize* = 5). Subsequently, 31,574 probe sets could be mapped to a unique genomic location with at least six perfect match probes (more than 50% of the 11 spotted probe-pairs per sequence). The probe sets in the expression matrix were annotated with the corresponding genes names; probe sets which could not be mapped to genes remain annotated with their original probe names. Further, to obtain a single expression level estimate per gene based on multiple probes the *collapseRow* function implemented in the WGCNA R package [48, 50, 83] was used to summarize the probe intensities. The resulting microarray expression matrix contained 33,204 genes or probe sets (i.e. where no mapping to genomic location was found).

To finally construct a genome wide rice co-expression network, the following approach was conducted: Initially, the pairwise similarities of all 33,204 genes or probe sets based on the expression profiles across the 29 tissues were quantified using PCC. Further, the approach developed by Langfelder and Horvath [50] is used to derive a *weighted* co-expression network. More specifically, a similarity matrix S was constructed in which the entry S_ij_ corresponds to the absolute value of the pairwise PCC:
1

where x_i_ and x_j_ represent of the expression profiles for genes or probe sets *i* and *j*,respectively.

Furthermore, the similarity matrix S was transformed into a weighted adjacency matrix, denoted by A. Here, the entry A_ij_ is obtained by raising the previously derived co-expression similarity S_ij_ to a power, β, β > =1:
2

The power, β, used to transform the similarity matrix is selected such that to the resulting network (described by its adjacency matrix) best approximates a scale-free topology – a defining network property of biological networks [84, 85]. In the case of the rice genome wide co-expression network, the parameter β = 7 was chosen (Additional file 10).

Gene modules were defined as sets of nodes in the co-expression network, i.e. genes and probe sets, with a high topological overlap [50, 51]. The topological overlap measure (TOM) between the i^th^ and j^th^ node is defined as
3

where  denotes the number of nodes to which both nodes i and j are connected by an edge,  denotes the sum of edge weights, i.e. the connection strengths, between i^th^ gene and the other genes. Further, 1-TOM denotes the TOM based dissimilarity measure (1-TOM) which was used for hierarchical clustering. Finally, gene modules are obtained by using dynamic tree cutting algorithm on the resulting dendrogram. This outlined procedure were carried out using the *blockwiseModule* method implemented in the WGCNA R package (parameters: maxBlockSize = 20000, power = 7, minModuleSize = 50, reassignThreshold = 0, mergeCutHeight = 0.20) [83].

### Connectivity scores of rice genes

Highly connected nodes in a network, commonly termed hubs, are thought to play a central role in the case of biological networks. The connectivity of a node has been used as a defining property for finding biologically relevant genes in co-expression networks [54]. Here, the intra-modular connectivity (kWithin) is used as a measure of centralization of genes. It is defined as the degree of the node corresponding to a gene inside a given module of the genome wide rice co-expression network [54]. The parameter kTotal was defined as the whole network connectivity for genes, reported as the sum of its connection strengths with all other genes in the network. A module’s eigengene-based connectivity (kME) was defined as the correlation between a gene expression value and the module eigengene (the average module expression value for an individual), which can be derived using R function *consensusKME* in the WGCNA package [50, 55].

### Plant material collection and cell wall composition determination

The 29 tissues, or organs, of Zhenshan 97, *indica* variety were harvested at 16:00–18:00 of the day according to Wang et al. [44]. All samples were dried at 50°C after inactivation at 105°C for 5 min. The dried tissues were ground through a 40 mesh screen and stored in a dry container until use.

Plant cell wall fractionation procedure and cell wall composition analysis were described by Peng et al. [56] with modification by Li et al. [58]. The crude cell wall material was suspended in 0.5% (w/v) ammonium oxalate and heated for 1 h in a boiling water bath (supernatant referred to as pectins). The remaining pellet was first re-suspended in 4 M KOH containing 1.0 mg/mL sodium borohydride for 1 h at 25°C., and then the combined supernatant was neutralized, dialyzed and lyophilized (referred to as hemicelluloses). The non-KOH-extractable residue defined as crude cellulose, was further extracted with acetic:nitric acids:water (8:1:2) for 1 h at 100°C, and the remaining pellet was defined as cellulose. Cellulose was analyzed by anthrone/H_2_SO_4_ method. Monosaccharides (xylose, arabinose, galactose) of hemicelluloses were determined by GC-MS [58].

Three monolignols were detected by HPLC [57]. All the samples were extracted with benzene:ethanol (2:1, v/v) in a Soxhlet for 4 h, the remaining pellet was then collected as cell wall residue (CWR). The procedure of nitrobenzene oxidation of lignin was carried out as follows: First, 0.05 g CWR was added with 5 mL 2 M NaOH and 0.5 mL nitrobenzene, and a stir bar was put into a 25 mL Teflon gasket in a stainless steel bomb, and the bomb was sealed tightly and heated at 170°C (oil bath) for 3.5 h and stirred at 20 rpm. Then, the bomb was cooled with cold water, the chromatographic internal standard (ethyl vanillin) was added to the oxidation mixture. To remove nitrobenzene and its reduction byproducts, the alkaline oxidation mixture was washed 3 times with 30 mL CH_2_CI_2_/ethyl acetate mixture (1/1, v/v).The alkaline solution was acidified to pH 3.0-4.0 with 6 M HCl, and then extracted with CH_2_CI_2_/ethyl acetate (3 × 30 mL) to obtain the lignin oxidation products which were in the organic phase. The organic extracts were evaporated to dryness under reduced pressure 40°C. Finally, the oxidation products were dissolved in 10 mL chromatographic pure methanol. All experiments were carried out in triplicate. Standard chemicals: p-Hydroxybenzaldehyde(H), vanillin(G) and syringaldehyde (S) were purchased from Sinopharm Chemical Reagent Co., Ltd.

### Identification of the cell wall-related modules through functional enrichment

GO terms of probes and genes were derived from agriGO [86]. To elucidate key biological processes, rather than conserved particular molecular functions, the GO sub-ontology 'biological process’ (GO-BP) was used for the gene-set enrichment analysis [87]. The enrichment analysis of particular GO-BP terms was performed using a weighted method in combination with Fisher’s exact test which is provided by topGO package [52]. KEGG ontology (KO) from the KEGG database (http://www.genome.jp/kegg/) [53] was additionally obtained and RAP IDs were converted to TIGR IDs using the RAP-DB ID converter tool (http://rapdb.dna.affrc.go.jp/tools/converter) [88]. KO enrichment was calculated by using hyper geometric test [89].

### Analysis of the cell wall-related modules through physiologic traits

For each gene module, the module eigengenes, *i.e.* the first principle component of the expression profiles of all the modules members, was derived as a representative expression profile for each module. Module eigengenes were calculated through the WGCNA R package [50, 83]. Subsequently, the association of module eigengenes and the measured physiological traits was determined as follows: for each module, the eigengene (ME) was tested for significant associations with the external traits. In case such an association is present, subsequently, a correlation analysis was performed between all of the modules genes and the cell wall components individually to study finer substructure of particular gene/external trait relationships. In addition to the degree of correlation to the trait, the genes intra-modular connectivity is used to rank putative gene candidates.

### Canonical correlations of cell wall traits and modules eigengenes

The set of cell wall features is represented by the matrix X in which rows correspond to the 29 tissues and columns correspond to the 7 cell wall measurements. Likewise, matrix Y of denotes the set of eigengenes whereas rows also correspond to the 29 tissues and columns correspond to the obtained 56 eigengenes. In CCA,  and  denote the two basis vectors, such that the correlation between the projections of the variables – columns in X and Y – onto these basis vectors given by  and  are mutually maximized: .

These derived linear projections *U*^1^ and *V*^1^ are called the first *canonical variates.* To investigate association between individual variables, i.e. eigengenes and cell wall features the similarity between variables in X and Y is quantified based on the Pearson correlations of their initial representation and the determined canonical variates. This form of correlations is known as canonical structure correlations [90] and can be further visualized by means of a relevance network [91]. Both, the CCA analysis as well the network are derived using the mixOmics package (http://www.math.univ-toulouse.fr/~biostat/mixOmics/) [92]. As a threshold for deriving edges between eigengenes and cell wall features in the relevance network, *τ*_*CCA*_ ≥ 0.5 for the absolute values of association between variables was chosen further ensuring that all 7 cell wall parameters are not isolated in this network.

### Availability of supporting data

All data sets supporting the results of this article are included within the article and also provided in the repository hosted by LabArchives, LLC (http://www.labarchives.com/) with DOI: http://dx.doi.org/10.6070/H4NV9G6V.

## Electronic supplementary material

Additional file 1: **The distribution of probes mapped to genes.** Columns in red enclosure indicate the different probes mapped to the same genes. Numbers of mapped probes are transformed as log. (PPT 110 KB)

Additional file 2: **Gene modules with rice locus identifiers, kME value of interested modules, kTotal, kWithin and kOut for each gene or probe set.** (XLS 8 MB)

Additional file 3: **Module eigenvector clustering and number of genes (probes) in each module.** A. Distribution of genes (probes) in each module, Red line indicated number of 500 genes. B. The co-expression network with 56 modules, and the eigenvectors of each module, calculated and clustered using the WGCNA software. (PPT 128 KB)

Additional file 4: **Gene ontology enrichment analysis of all modules at** ***p*** **< 0.05.** (XLS 394 KB)

Additional file 5: **KEGG enrichment analysis of all gene co-expression modules at** ***p*** **< 0.05.** (XLS 200 KB)

Additional file 6: **Genes/Probes and their orthologs involved in Module 44, 24, 40, 42, 46 with all k-values (kTotal,kWithin kOut and kME except 6B) and predict function were listed.** Ara:Arabinose related function; Gal:Galatose synthesis;Multi:Mutile roles;NA: None mapped;NF:None ortholog genes. (XLS 226 KB)

Additional file 7: **Variation of cell wall components in rice.** (PDF 150 KB)

Additional file 8: **Correlation coefficient values between cell wall components.** (PDF 25 KB)

Additional file 9: **MapMan ontology term enrichment for Module 34 and 42 genes.** (XLS 64 KB)

Additional file 10: **Analysis of network topology through different soft-thresholding powers.** Left panel displays the scale-free fit index as a function of the soft-thresholding power. Right panel shows the mean connectivity (degree) as a function of the soft-thresholding power. (PPT 62 KB)
