# Supplementary figures and images for: An integrated genomic and metabolomic framework for cell wall biology in rice
Source: BMC Genomics. 2014 Jul 15;15(1):596. doi: 10.1186/1471-2164-15-596 (PMC4112216; doi:10.1186/1471-2164-15-596)

## Slide 1
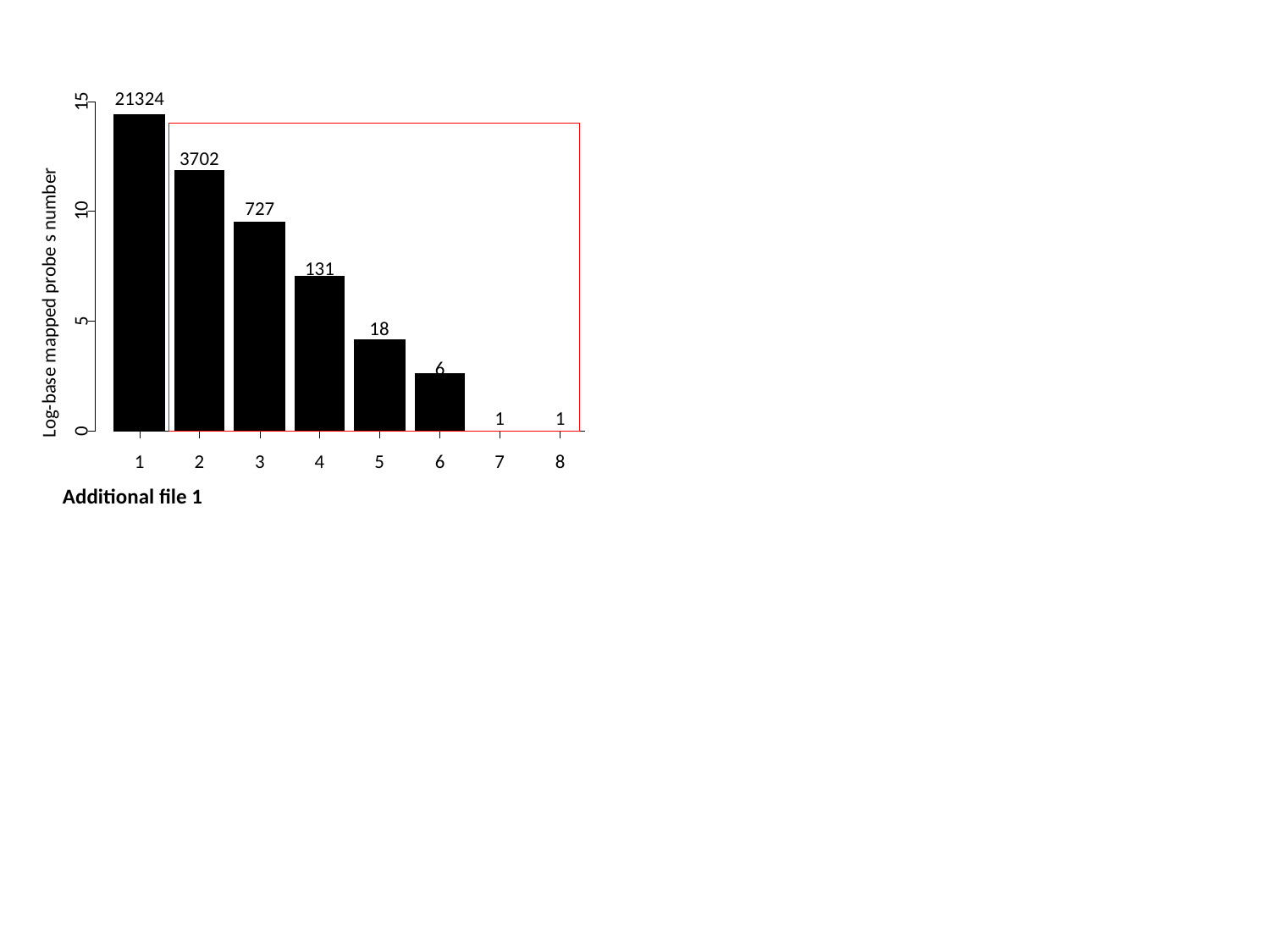

21324
15
3702
727
10
131
Log-base mapped probe s number
5
18
6
1
1
0
1
2
3
4
5
6
7
8
Additional file 1

Supplement: Supplementary file 1 — Additional file 1: The distribution of probes mapped to genes. Columns in red enclosure indicate the different probes mapped to the same genes. Numbers of mapped probes are transformed as log. (PPT 110 KB) [file 12864_2013_6285_MOESM1_ESM.ppt]

## Slide 1
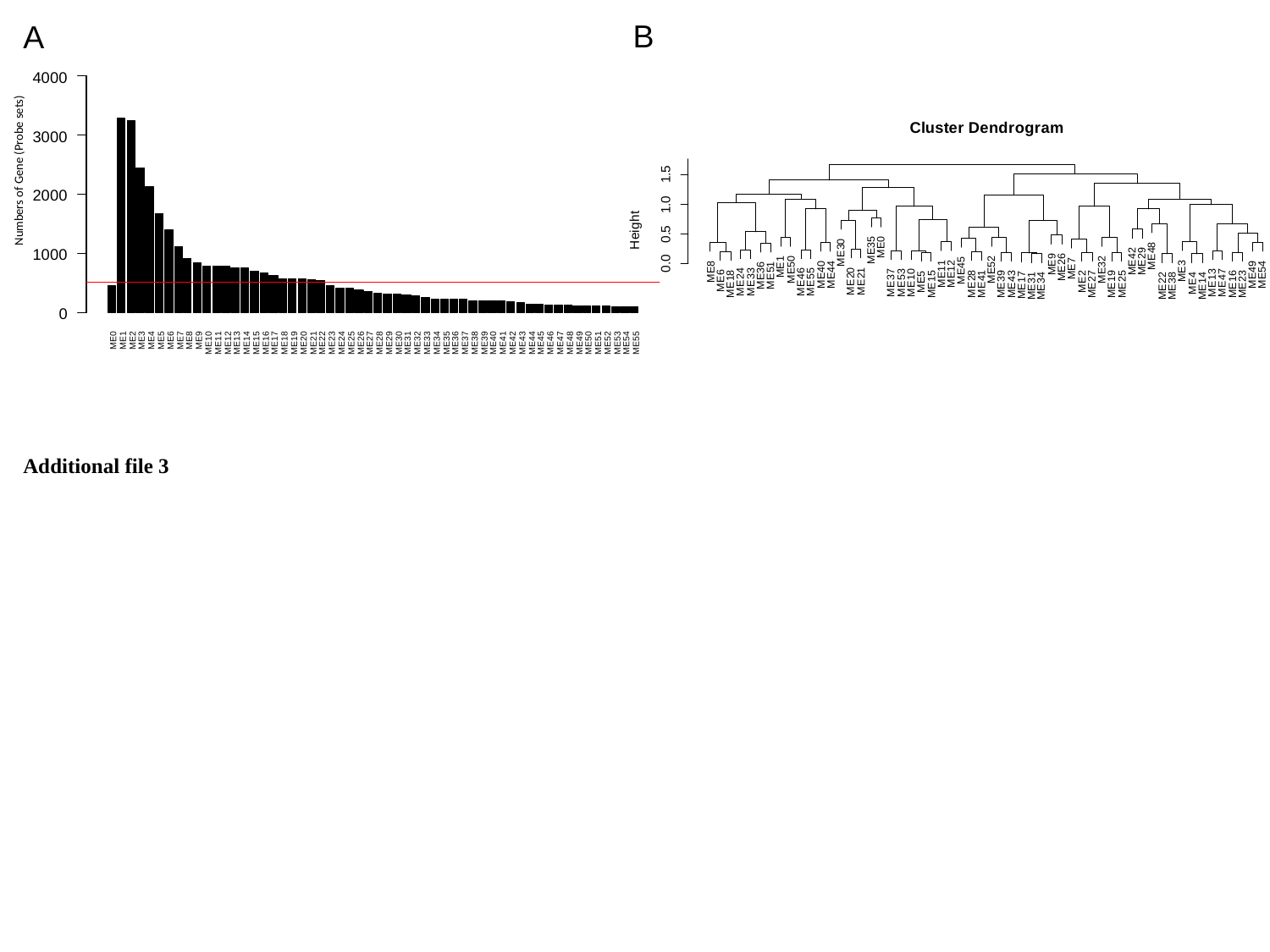

B
A
Numbers of Gene (Probe sets)
Additional file 3

Supplement: Supplementary file 3 — Additional file 3: Module eigenvector clustering and number of genes (probes) in each module. A. Distribution of genes (probes) in each module, Red line indicated number of 500 genes. B. The co-expression network with 56 modules, and the eigenvectors of each module, calculated and clustered using the WGCNA software. (PPT 128 KB) [file 12864_2013_6285_MOESM3_ESM.ppt]

## Slide 1
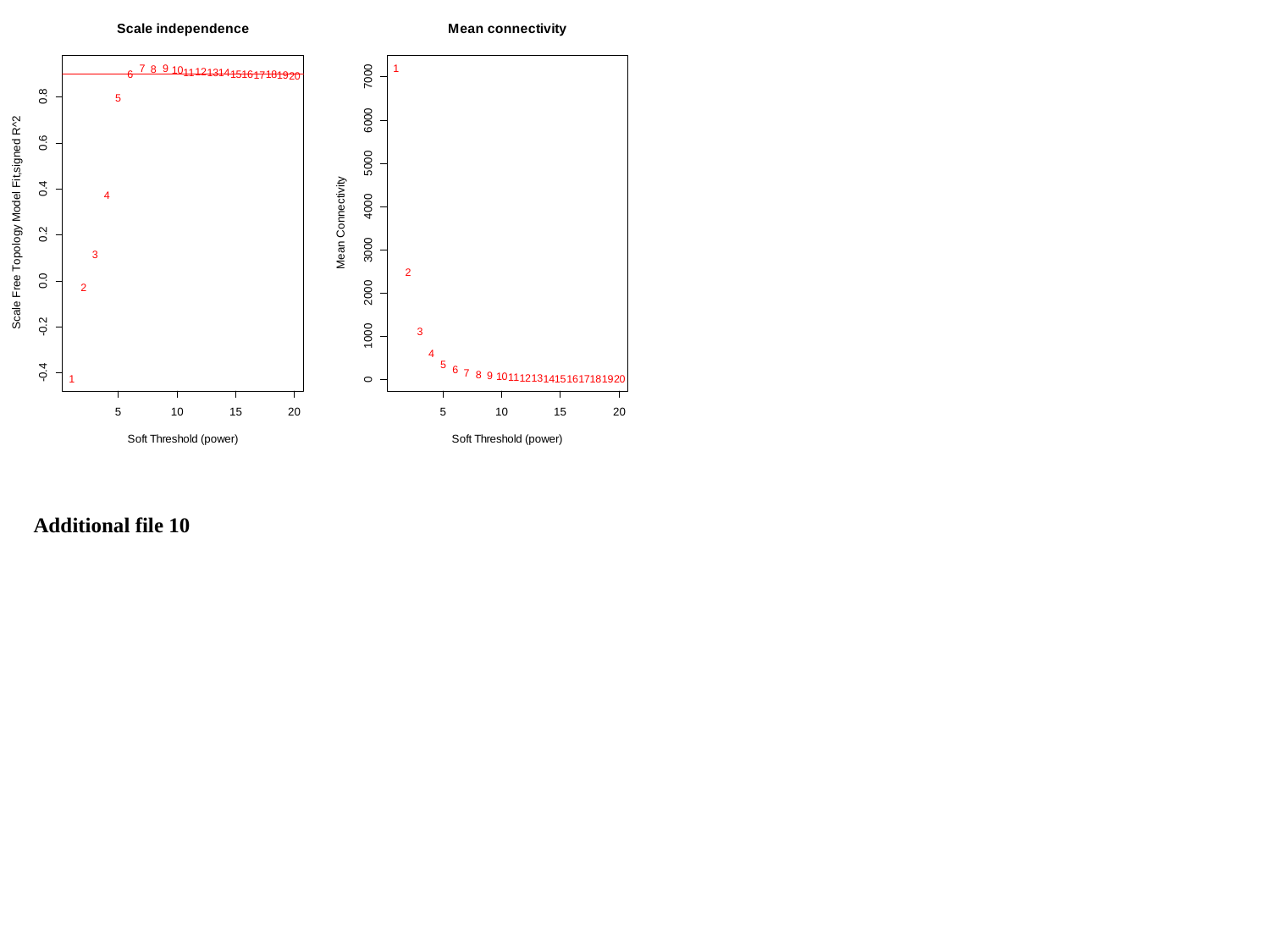

Additional file 10

Supplement: Supplementary file 10 — Additional file 10: Analysis of network topology through different soft-thresholding powers. Left panel displays the scale-free fit index as a function of the soft-thresholding power. Right panel shows the mean connectivity (degree) as a function of the soft-thresholding power. (PPT 62 KB) [file 12864_2013_6285_MOESM10_ESM.ppt]
