# Supplementary material for: An integrated genomic and metabolomic framework for cell wall biology in rice
Source: BMC Genomics. 2014 Jul 15;15(1):596. doi: 10.1186/1471-2164-15-596 (PMC4112216; doi:10.1186/1471-2164-15-596)
Supplement: Supplementary file 7 — Additional file 7: Variation of cell wall components in rice. (PDF 150 KB) [file 12864_2013_6285_MOESM7_ESM.pdf]

Tissues(organs)	Cellulose (% dry matter)	Arabinose (mg/g)	Xylose (mg/g)	Galactose (mg/g)	H(umol/g)	G(umol/g)	S(umol/g)
**Callus**	5.31(0.27)*	41.07(4.17)	68.07(10.58)	14.81(0.69)	20.01(1.13)	54.98(4.63)	6.72(1.00)
**Callus/Subculture**	8.69(0.34)	26.31(1.86)	23.02(1.21)	7.08(0.68)	20.49(0.80)	33.61(1.55)	6.84(0.56)
**Germinating seed**	5.68(0.33)	6.14(0.48)	24.60(1.33)	1.29(0.26)	17.21(1.45)	28.63(2.06)	3.00(0.22)
**Seed germination**	8.89(0.30)	11.74(1.34)	47.41(1.47)	2.34(0.13)	19.33(2.57)	51.82(2.22)	8.53(0.93)
**Plumule/Dark**	5.73(0.40)	20.67(1.10)	26.95(3.91)	5.17(1.51)	12.31(0.16)	16.60(1.47)	2.37(0.33)
**Plumule/Light**	15.77(0.28)	19.35(3.89)	61.14(14.48)	3.75(0.91)	16.19(2.25)	10.18(7.02)	8.85(0.76)
**Radicle/Dark**	15.49(0.43)	20.60(1.20)	59.41(5.75)	11.22(0.67)	17.44(0.49)	18.81(4.95)	3.57(1.14)
**Radicle/Light**	17.72(0.68)	22.22(0.49)	61.05(5.13)	9.25(0.37)	14.94(0.36)	29.29(1.23)	9.04(0.82)
**Seedlings**	16.57(0.23)	28.29(3.27)	125.53(20.62)	7.51(1.10)	23.21(9.01)	44.06(13.05)	12.13(4.63)
**Young shoot**	13.82(0.08)	14.24(1.77)	63.98(11.61)	2.27(0.15)	21.32(0.81)	29.73(0.63)	5.33(0.15)
**Young Root**	19.49(0.53)	28.43(0.64)	91.87(4.25)	8.48(0.36)	50.87(2.10)	62.15(1.90)	25.12(0.08)
**Mature Leaf**	15.14(0.40)	15.27(1.11)	79.60(9.01)	2.35(0.73)	29.39(2.54)	34.46(1.75)	6.82(0.49)
**Old leaf**	19.19(0.62)	17.60(1.18)	78.86(14.60)	2.60(0.27)	25.67(1.73)	40.80(4.52)	11.10(1.10)
**Mature sheath**	15.57(0.27)	14.95(0.92)	79.57(8.27)	1.71(0.46)	17.24(3.32)	28.90(5.25)	6.88(1.33)
**Old sheath**	20.06(0.51)	16.12(0.74)	106.52(0.49)	1.90(1.08)	21.77(0.91)	40.70(1.95)	12.57(1.69)
**Young Flag leaf**	13.54(0.09)	15.83(0.47)	96.56(11.64)	1.38(0.09)	30.18(10.34)	36.61(3.99)	7.26(0.27)
**Old flag leaf**	11.69(0.51)	16.39(0.66)	108.49(4.55)	2.59(0.07)	31.12(4.57)	57.83(10.61)	21.45(3.62)
**Panicle 2**	2.05(0.04)	8.89(1.05)	12.87(0.27)	3.26(0.29)	14.19(0.98)	2.45(0.63)	7.65(1.44)
**Panicle 3**	1.99(0.10)	9.39(1.66)	12.92(1.55)	3.44(0.43)	20.95(5.66)	48.54(3.54)	9.76(3.49)
**Young Panicle**	2.77(0.08)	9.56(1.08)	14.24(2.91)	3.62(1.01)	13.62(0.42)	1.41(0.43)	9.15(1.67)
**Old Panicle**	25.23(0.46)	19.85(1.92)	222.56(27.23)	1.76(0.18)	63.88(9.79)	73.20(3.93)	26.19(0.59)
**Young Stem**	6.01(0.13)	10.20(0.05)	63.91(3.69)	1.18(0.47)	30.00(10.04)	41.13(11.60)	13.69(4.12)
**Old Stem**	18.85(0.45)	12.64(1.20)	113.56(18.66)	1.64(0.29)	43.07(0.90)	48.58(0.25)	25.42(0.11)
**Palea/lemma**	31.33(0.90)	19.06(0.88)	245.82(21.39)	1.61(0.07)	71.72(3.21)	54.50(3.72)	25.18(0.63)
**Spikelet**	26.86(0.42)	16.58(0.81)	177.87(22.58)	1.88(0.13)	65.49(8.27)	107.19(4.80)	22.47(3.39)
**Stamen**	2.30(0.02)	21.78(1.82)	30.76(5.25)	3.49(0.66)	13.63(0.31)	17.36(0.10)	4.80(0.27)
**Endosperm 1**	0.29(0.03)	3.26(0.47)	3.49(0.73)	0.03(0.03)	4.93(0.41)	1.18(0.09)	1.07(0.14)
**Endosperm 2**	0.67(0.03)	3.98(1.19)	5.83(1.63)	0.16(0.03)	8.09(1.27)	1.60(0.56)	1.06(0.36)
**Endosperm 3**	0.68(0.07)	5.71(1.11)	6.93(1.51)	1.12(0.75)	7.12(0.21)	1.35(0.08)	1.07(0.08)
