# Supplementary material for: An integrated genomic and metabolomic framework for cell wall biology in rice
Source: BMC Genomics. 2014 Jul 15;15(1):596. doi: 10.1186/1471-2164-15-596 (PMC4112216; doi:10.1186/1471-2164-15-596)
Supplement: Supplementary file 8 — Additional file 8: Correlation coefficient values between cell wall components. (PDF 25 KB) [file 12864_2013_6285_MOESM8_ESM.pdf]

	Cellulose	Arabinose	Xylose	Galatcose	H unit	G unit	S unit
**Cellulose**		0.37*	0.89**	0.03	0.81**	0.70**	0.71**
**Arabinose**	0.37*		0.34	0.81**	0.26	0.42*	0.25
**Xylose**	0.89**	0.34		-0.06	0.90**	0.75**	0.81**
**Galatcose**	0.03	0.81**	-0.06		-0.10	0.09	-0.06
**H unit**	0.81**	0.26	0.90**	-0.10		0.81**	0.89**
**G unit**	0.70**	0.42*	0.75**	0.09	0.81**		0.76**
**S unit**	0.71**	0.25*	0.81**	-0.06	0.89**	0.76**	

* $p < 0.05$;   ** $p < 0.01$.
